# A Compelling Case of Autoimmune Hemolytic Anemia and Its Potential Association With SARS-CoV-2

**DOI:** 10.7759/cureus.39566

**Published:** 2023-05-27

**Authors:** José Miguel Silva, Joana Gomes Cochicho, Armando Cruz Nodarse, Isabel Lavadinho

**Affiliations:** 1 Internal Medicine, Hospital Doutor José Maria Grande, Portalegre, PRT

**Keywords:** autoimmune hemolytic anemia (aiha), aiha, hemolytic anaemia, sars-cov-2, covid 19

## Abstract

Autoimmune hemolytic anemia can be caused by infections, lymphoproliferative disorders, autoimmune disorders, or triggered by drugs or toxins.

We present the case of a 92-year-old man admitted with gastrointestinal symptoms. He presented with autoimmune hemolytic anemia. The etiologic study was negative for autoimmune conditions or solid masses. Viral serologies were negative, and RT-PCR for SARS-CoV-2 was positive. The patient began treatment with corticoid, with resulted in cessation of hemolysis and improvement of the anemia.

A few cases of autoimmune hemolytic anemia have been reported in COVID-19 patients. In this case, the infection seems to coincide with the hemolysis period, and we found no other cause for this event. So, we highlight the need to search for SARS-CoV-2 as a possible infective cause of autoimmune hemolytic anemia.

## Introduction

Hemolytic anemia is caused by the destruction of red blood cells, leading to an increase in hemoglobin catabolism, a decrease in hemoglobin levels, and an increase in erythroid production effort in the bone marrow [1].

The causes of hemolytic anemia can be acute or chronic and can include membrane protein deficiencies, microangiopathies, increased oxidative stress, and antibodies against red blood cells, among others [1].

When hemolytic anemia is mediated by self-antibodies, it's referred to as autoimmune hemolytic anemia (AIHA) and can be classified as idiopathic or secondary. The latter group includes causes such as infectious agents, lymphoproliferative diseases, autoimmune diseases, and drugs or toxins [2].

AIHA has been reported in the context of COVID-19, mainly in patients with comorbidities such as thrombocytopenia, leukemia, lymphoma, various cancers, and autoimmune conditions [3].

## Case presentation

We present the case of a 92-year-old male with medical records of chronic ischemic heart disease, hypertension, dyslipidemia, and type 2 diabetes mellitus. There wasn’t any previous history of hemolytic anemia. The patient was chronically medicated with clopidogrel, metformin, perindopril, indapamide, amlodipine, simvastatin, carvedilol, and furosemide. There had not been any recent drug introductions.

All of the family members developed SARS-CoV-2 infections, but none of them developed anemia. 

The patient was seen in the emergency department with a chief complaint of abdominal pain, vomiting, and fever. He had not taken any relief medication before coming to the hospital. Physical examination showed abdominal tenderness without defense, slight tachycardia, a blood pressure of 95/62 mmHg, and a temperature of 39.2°C. No respiratory distress was evident, and lung sounds were normal. The cardiac auscultation was rhythmic without murmurs. The patient presented with skin pallor without cyanosis or acrocyanosis.

From the analytical evaluation to admission, we highlight a hemoglobin of 6.7 g/dL and a positive real-time polymerase chain reaction for SARS-CoV-2. The patient had a hemoglobin of 11.9 g/dL before the event.

Support treatment for the viral infection was started, and given the ischemic heart disease, a blood transfusion was performed. The patient developed a transfusion-related acute lung injury, which required suspension of the transfusion and symptom relief. The administration of immunomodulation agents was not necessary.

The SARS-CoV-2 infection manifested itself only with slight gastrointestinal symptoms, and the patient was transferred to the Internal Medicine Department for further study of the anemia.

Analytically, there was a slight decrease in the hemoglobin level to 5.5 g/dL. The erythrocytes presented an elevated medium corpuscular volume (MCV) and an elevated red cell distribution width (RDW). The peripheral blood smear confirmed macrocytic and normochromic anemia, marked anisopoikilocytosis, and polychromasia. Vitamin deficiencies were excluded. The folic acid was 7.05 ng/mL (reference 4.6 to 18.0), and the vitamin B12 was 307 pg/mL (reference 197 to 770).

The patient also presented an elevated lactate dehydrogenase (LDH) of 883 U/L, reduced haptoglobin (10 mg/dL), elevated bilirubin, and positive direct and indirect antiglobulin tests performed at body temperature (Table 1).

**Table 1:** Analytic evolution MCV: mean corpuscular volume, RDW: red cell distribution width, CK: creatine kinase

	Reference values	Day 1	Day 3	Day 4	Day 5	Day 8	Day 12	Day 16	Day 20	Day 24	Day 27	Day 53
Erythrocytes (x10^6/ µL)	4.3-6.4	1 510	1 580	1 280	1 270	1 570	1 800	1 910	2 040	2 250	2 160	3 660
Hemoglobin (g/dL)	13.5-18.0	6.7	6.6	5.5	5.2	6.5	7.3	7.6	7.8	8.4	8.1	13.3
MCV (fL)	80.0-97.0	131.1	127.8	126.6	127.6	127.4	126.1	122.5	119.6	118.2	116.2	103.0
RDW (%)	11.5-15.0	19.4	19.9	16.3	16.7	19.8	18.4	16.6	15.5	15.1	15.6	14.7
Reticulocytes (x10^3/ µL)	0.5-2.0				2 00	32 0	420	260	300	210	230	
Leukocytes (x10^3 /µL)	4.0-11.0	13 180	9 240	13 780	13 320	16 510	14 410	12 430	8 110	6 330	6 860	8 250
Platelets (x10^3/µL)	140 000-440 000	237 000	223 000	191 000	192 000	276 000	405 000	384 000	268 000	220 000	195 000	186 000
Lactate dehydrogenase (U/L)	135-220	766	918	883	913	838	570	337	256	226	182	291
Total bilirubin (mg/dL)	0.0-1.2	3.06	2.98	2.42	2.8	2.95	2.63		1.53	1.57	1.36	
Direct bilirubin (mg/dL)	0.0-0.2	0.66	0.84	1.03	1.31	1.32	1.33		0.83	0.72	0.71	
CK (U/L)	0-190	320	1835	872		65	17					
Direct antiglobulin test				Positive								
Indirect antiglobulin test				Positive								
Haptoglobin (mg/dL)					10.0						91.0	
C-reactive protein (mg/L)	0.0-5.0		39.9	80.2			26.9					11.4

These analytical characteristics supported the diagnosis of AIHA by warm antibodies. The patient started treatment with prednisolone (1 mg/kg/day) and supplementation with folic acid. After 27 days, there was a normalization of LDH and improvement of hemoglobin values, and the patient started reducing the corticoid dose.

As an intercurrence, the patient presented with decompensation of diabetes due to corticosteroid therapy, leading to the need for insulin therapy.

Concerning the etiological investigation, serologies for cytomegalovirus, Epstein-Barr virus, coxsackie virus, parvovirus B19, hepatitis C virus, and mycoplasma were negative. The anti-dsDNA antibody was negative, and the screening for 17 autoantibodies associated with connective tissue diseases was also negative. Computed tomography of the chest, abdomen, and pelvis did not reveal any solid masses.

After 32 days, the patient was discharged, maintaining his corticoid weaning, insulin therapy, and folic acid supplementation. On day 53, he presented with 13.3 g/dL of hemoglobin and normal LDH and haptoglobin.

## Discussion

Although a rare condition recently, an increasing number of AIHA secondary to COVID-19 infections in immunocompetent patients has been described worldwide [3-6]. However, the exact mechanism is not determined. 

Concerning SARS-CoV-2, its cellular infectious process involves the interaction with the angiotensin-converting enzyme 2 receptors, CD147 protein, and erythrocyte Band3 protein. A possible mechanism for immune hemolytic anemia in these patients is the formation of autoantibodies through CD147 protein or erythrocyte Band3 protein [2].

Another mechanism was hypothesized, based on molecular mimicry, between the spike protein of the SARS-CoV-2 surface and the Ankyrin 1 protein, which is an erythrocyte membrane protein that provides a connection between the membrane skeleton and the plasma membrane. These proteins present a similar immunogenic‐antigenic epitope already identified [7]. 

In most cases of autoimmune hemolytic anemia triggered by infections, the process results from the formation of cold antibodies of the immunoglobulin M (IgM) type, which bind to the RBC membrane and activate the complement, leading to intravascular hemolysis or phagocytosis in the extravascular space [8]. On the other hand, in warm AIHA, the process involves the opsonization of erythrocytes with immunoglobulin G (IgG) and subsequent destruction by the mononuclear phagocytic system in the spleen (extravascular hemolysis) [9].

Concerning SARS-CoV-2 infection, to date, case reports have associated COVID-19 with the formation of either warm or cold autoimmune antibodies [8]. There were also reports of the simultaneous identification of both antibody types [9].

In our case, direct and indirect Coombs tests were performed at body temperature, which confirms the presence of warm antibodies. Unfortunately, due to technical difficulties, the tests were not performed at a cold temperature. So, we cannot confirm or exclude the existence of this mechanism. 

## Conclusions

In the presented case, the onset of AIHA coincident with the infection, and the absence of other clinical or analytical factors that justify hemolysis suggests that SARS-CoV-2 played an important role in triggering the event.

SARS-CoV-2 is currently an endemic virus that integrates a vast group of viruses responsible for triggering respiratory infections. This clinical case is meant to alert healthcare professionals to this possible etiological agent in cases of AIHA. The reporting of this event is also important for future research to build a series of cases and consolidate existing knowledge about pathological mechanisms.
